# Posterior Capsule Opacification after Cataract Surgery via Implantation with Hydrophobic Acrylic Lens Compared with Silicone Intraocular Lens: A Systematic Review and Meta-Analysis

**DOI:** 10.1155/2022/3570399

**Published:** 2022-02-25

**Authors:** Ye Rin Kwon, Yoo Na Hwang, Sung Min Kim

**Affiliations:** ^1^Department of Medical Devices Industry, Dongguk University-Seoul (04620), 26 Pil-dong 3-ga Jung-gu, Seoul, Republic of Korea; ^2^Department of Medical Biotechnology, Dongguk University-Biomedical Campus (10326), 32 Dongguk-ro Ilsandong-gu, Goyang-si, Gyeonggi-do, Republic of Korea

## Abstract

Hydrophobic acrylic intraocular lens (IOL) is the most popular material in cataract surgery. Posterior capsule opacification (PCO) is a long-term complication of cataract surgery. It can impair vision and adversely affect the prognosis of IOL delamination. The objective of this study was to perform a systematic review and meta-analysis to provide an updated evaluation of long-term complications and visual function after implantation with hydrophobic acrylic and silicone intraocular lenses. PubMed, Embase, and Cochrane Library were searched from January 2000 until March 2021. Randomized controlled trials (RCTs) and retrospective studies were finally included. The main outcomes were PCO value and neodymium-doped yttrium aluminum garnet (Nd : YAG) capsulotomy rate. Subgroup analysis was performed to compare hydrophobic acrylic and silicone IOLs during the follow-up period. Sensitivity analysis was also performed. The meta-analysis included a total of 17 studies. When the follow-up period was considered, the results of the analysis revealed higher PCO value (Group 3: standardized mean difference (SMD), −0.59; 95% confidence interval (CI), −0.90 to −0.28) and Nd : YAG capsulotomy rate (Group 3: risk ratio (RR), 0.60; 95% CI, 0.40–0.89) for hydrophobic acrylic IOLs than silicone IOLs during a long-term (≥6 years) follow-up. In conclusion, both the PCO value and the Nd : YAG capsulotomy rates were higher in hydrophobic acrylic IOLs group than the silicone IOLs group at long-term use (more than 6 years) after implantation.

## 1. Introduction

Cataract surgery is frequently performed worldwide primarily due to aging [1, 2]. Cataract is prevalent in adults aged between 45 and 50 years [3]. Statistical data pertaining to cataracts in the United States have reported a prevalence of nearly 32% among adults below the age of 65 years and 50% among those in their 40s and 50s [4]. Age-related cataract surgery is also being performed earlier than before [5]. As a result, long-term safety and efficacy of intraocular lens (IOL) implantation have been established [6–8].

Materials of IOLs can be distinguished by their moisture content, chemical composition, refractive index, and tensile strength. Differences in these properties can determine complications and vision [9]. Posterior capsule opacification (PCO) value and neodymium-doped yttrium aluminum garnet (Nd : YAG) capsulotomy rate are typical indicators of the incidence of complications after cataract surgeries [10]. In particular, PCO is a representative long-term complication following cataract surgery. It is caused by fibrosis around the posterior capsule [11–13]. This PCO can lead to impaired vision, contrast sensitivity, and glare [11, 13]. PCO can be easily treated via Nd : YAG capsulotomy [10, 12]. However, Nd : YAG capsulotomy can increase the risk of IOL instability, dislocation, or further complications such as increased intraocular pressure, glaucoma, retinal detachment, and cystic macular edema [10, 14, 15].

Hydrophobic acrylic IOLs are widely used because they can reduce complications such as PCO and optimize vision [10, 16]. Theoretically, hydrophobic acrylic IOLs in bioactive materials are known to prevent serious PCO compared to IOLs in polymethyl methacrylate (PMMA) or silicone materials [17–19]. Several studies [18, 19] have reported that hydrophobic acrylic IOLs can yield a lower PCO value than hydrophilic acrylic IOLs. However, clinical studies [7, 8], including long-term follow-up (over six years), have demonstrated that hydrophobic acrylic IOLs are associated with a relatively higher PCO value or Nd : YAG capsulotomy rate than silicone IOLs. In particular, Rønbeck and Kugelberg [7] have reported a higher degree of survival without Nd : YAG capsulotomy in a 12-year follow-up analysis of silicone IOLs compared with hydrophobic acrylic IOLs at more than 6 to 7 years after cataract surgery. Cheng et al. [20] have stated that clinical trials lasting at least five years are needed to further evaluate the impact of IOL materials on PCO reduction and the use of Nd : YAG capsulotomy. Therefore, we conducted a systematic review and meta-analysis to determine whether hydrophobic acrylic IOLs after cataract surgery might be more effective than silicone IOLs in reducing postsurgical complications during a long-term follow-up.

## 2. Methods

### 2.1. Literature Search

This review was conducted following the updated Preferred Reporting Items for Systematic reviews and Meta-Analysis (PRISMA) 2020 statement [21] (PRISMA 2020 checklist is detailed in Supplementary File 1). This study was registered with the International Prospective Register of Systematic Reviews (PROSPERO) database (identifier: CRD42021242394). Reports of randomized controlled trials (RCTs) and retrospective studies comparing hydrophobic acrylic IOLs with silicone IOLs in patients with age-related cataracts were identified via a systematic search of PubMed, Embase, and Cochrane Library. The search period was extended from January 2000 to March 2021 to cover long-term follow-up studies. Search terms used a combination of MeSH/Emtree terms and “natural language terminology,” including cataract, intraocular lens, lens implantation, capsule opacification, hydrophobic acrylic, and silicone (search strategy is detailed in Supplementary File 2). In case of duplicate studies with data extracted from the same population group, only the most recent studies were included. Any disagreements regarding the search strategy were resolved via consensus based on discussion.

### 2.2. Selection Criteria

Studies fulfilling the following selection criteria were included: (1) patients >45 years of age who had age-related cataract and treated with cataract surgery; (2) interventions using hydrophobic acrylic IOLs; (3) comparison with silicone IOLs; (4) outcomes included at least one of the following outcome variables: PCO value, Nd : YAG capsulotomy rate, visual acuity, anterior capsule opacification (ACO) value, tilt, and decentration; (5) RCTs and retrospective studies. Case studies, pilot studies, grey literature, studies published in languages other than English, patients with congenital or traumatic cataracts, and diabetes requiring medical control were excluded from this study. Studies were selected by two reviewers (Y.R. and Y.N.). The first reviewer (Y.R.) reviewed all titles, abstracts, and full texts. The second reviewer (Y.N.) analyzed studies excluded from the review.

### 2.3. Data Extraction and Quality Assessment

The following data were extracted from each study: author's name, year of publication, study design, number of eyes, patient's age and gender, follow-up period, IOL materials and designs, and individual study outcomes. Primary outcomes were set at the quantitative PCO value represented by score or grade using evaluative software and Nd : YAG capsulotomy rate to compare the degree of postsurgical complications during the long-term follow-up after cataract surgery. Secondary outcomes were ACO value represented by score or grade or area, visual acuity (best-corrected visual acuity, BCVA) represented by the logarithm of the minimum angle of resolution (log MAR), degree of tilt, and decentration in relation to complications immediately following cataract surgery or visual function. PCO values and Nd : YAG capsulotomy rates were determined and categorized according to the follow-up period. In the case of multiple values, all values that could be included in a subgroup were extracted. In other cases, only the most recent values were extracted. Quality assessment of included RCT studies was performed using the Cochrane group's Risk of Bias (ROB) tool [22]. Retrospective studies were assessed using the Risk of Bias In Nonrandomized Studies–of Interventions (ROBINS-I) tool [22]. All controversies were resolved via consensus based on discussion among reviewers.

### 2.4. Data Analysis

Sensitivity analysis was performed except for studies with missing SD data. According to Cochrane's handbook [22], missing SDs were replaced with the mean value of SD based on values determined using the same evaluative system. PCO and ACO values with various measurement scales as continuous variables were pooled using standardized mean differences (SMDs) with 95% confidence intervals (CIs). Dichotomous variables of Nd : YAG capsulotomy rate were calculated using relative risks (RRs) with 95% CIs. Outcomes of visual acuity, tilt, and decentration were pooled using mean differences (MDs) with 95% CIs. Meta-analysis was considered statistically significant if *P*-value was less than 0.05. For heterogeneity, *I*^2^ values greater than 75% represented high heterogeneity [23] using a random-effects model. Publication bias was visually evaluated via funnel plots. All data analyses for the meta-analysis were performed using RevMan (version 5.4.1, Cochrane Library).

### 2.5. Subgroup Analysis

Subgroup analysis was performed to confirm results according to the follow-up period. Based on the study of Rønbeck and Kugelberg [7], the following three groups were created according to the length of the follow-up period: (1) Group 1 (G1), short term, 0 years ≤ follow-up period < 3 years; (2) Group 2 (G2), medium term, 3 years ≤ follow-up period < 6 years; (3) Group 3 (G3), long term, follow-up period ≥ 6 years.

## 3. Results

### 3.1. Included Studies

**Figure 1:**
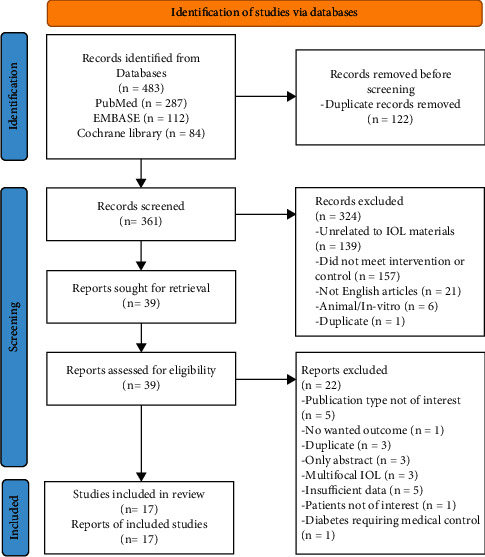
PRISMA flow diagram outlining study selection.

A total of 483 articles were identified in the initial analysis. Of them, 122 duplicated articles were excluded. Based on titles and abstracts, 39 potential studies were screened. Finally, 17 eligible studies [6–8, 24–37] were included in this analysis (excluded studies and reasons for exclusion are detailed in Supplementary File 3). The flow diagram of the selection process is presented in Figure 1.

### 3.2. Characteristics of the Included Studies

Characteristics of included studies are listed in Table 1. This meta-analysis included 14 RCTs [7, 8, 25, 27–37] and three retrospective studies [6, 24, 26]. The average age of patients ranged from 61.3 to 78 years. The follow-up period varied from one week to 12 years. A subgroup analysis was performed to determine PCO values and Nd : YAG capsulotomy rates. These subgroups were separated by follow-up periods. Based on PCO values, four studies [8, 27, 28, 34] were included in the short-term group (G1), seven studies [8, 24, 26, 27, 31, 36, 37] were included in the medium-term group (G2), and two studies [6, 8] were included in the long-term group (G3). Based on Nd : YAG capsulotomy rates, the short-term group (G1) included four studies [24, 28, 33, 34], the medium-term group (G2) comprised six studies [24, 27, 29, 31, 32, 37], and the long-term group (G3) had three studies [6–8]. These included studies were conducted in the Netherlands, Germany, United States, Austria, Japan, South Korea, Finland, Italy, Sweden, and Lithuania (characteristics of IOLs included in the meta-analysis are detailed in Supplementary File 4).

### 3.3. Assessment of Risk of Bias

**Figure 2:**
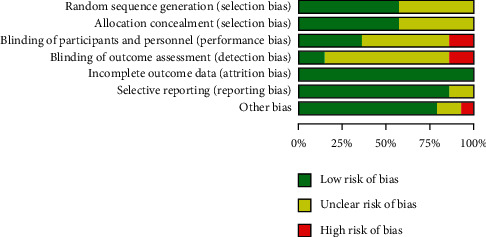
Risk of bias assessment of RCTs.

**Figure 3:**
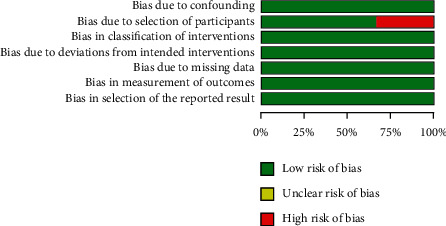
Risk of bias assessment of non-RCTs.

Figure 2 summarizes the risk of bias in 14 RCTs using the ROB tool. Investigators used an envelope [28, 30], a randomization scheme [25], or a computerized random number generator [7, 8, 27, 29, 31] for random assignment of the 14 RCTs included in the present meta-analysis. Of these 14 RCTs, five [8, 27–29, 35] were double-blind, one study [25] was single-blind, and two studies [31, 37] were impossible to blind. In the case of single-blind or nonblinded trials, the risk of performance bias was deemed high. When random assignment and blinding methods were not specified, they were considered to have an unclear risk.  Figure 3 summarizes the risk of bias in three retrospective studies using the ROBINS-I tool. Patients were recruited by follow-up visits [24, 26] or invitations [6] of patients conducted by the same surgeon. The risk of participant selection bias was deemed high when only patients who met the preliminary criteria were recruited by the same surgeon retrospectively [26]. All studies reported the number and reason of dropout patients (bias of each study is detailed in Supplementary Files 5 and 6).

### 3.4. Comparison of the Degree of Complications Based on Long-Term Follow-Up after Cataract Surgery

#### 3.4.1. PCO Value

**Figure 4:**
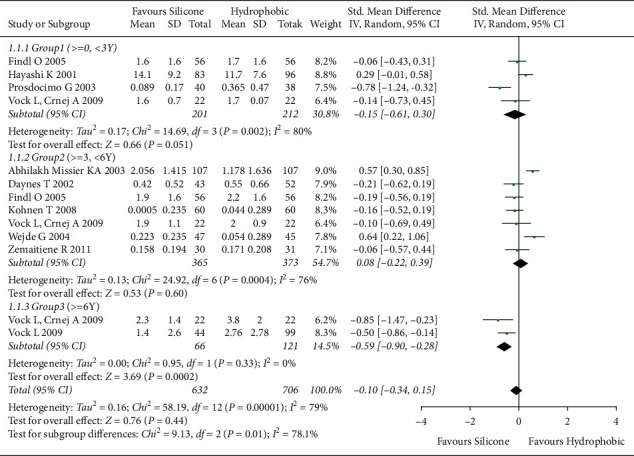
Comparison of subgroup effects on PCO value between hydrophobic acrylic and silicone IOLs. In the long-term group (G3, follow-up period ≥6 years), hydrophobic acrylic IOLs showed significantly high PCO values than silicone IOLs. Chi^2^ = chi-square statistic; CI = confidence interval; df = degrees of freedom; *I*^2^ = I-squared, heterogeneity statistic; IOL = intraocular lens; IV = inverse variance; PCO = posterior capsule opacification; SMD = standard mean difference; *Z* = Z-statistic.

PCO values of hydrophobic acrylic and silicone IOLs were comparatively analyzed in 10 studies [6, 8, 24, 26–28, 31, 34, 36, 37] comprising 1,138 eyes. A random-effects model was used due to the high heterogeneity (*I*^2^ = 80%) of studies. The overall effect on PCO value showed no statistically significant difference between hydrophobic acrylic and silicone IOLs when the follow-up period was not considered ([SMD], −0.23; 95% CI, −0.50 to 0.05; *P*=0.11). The forest plot is detailed in Supplementary File 7. Subgroup analysis during the follow-up period revealed a high heterogeneity (*I*^2^ = 79%). Therefore, the random-effects model was used. Short-term (G1, 0 years ≤ follow-up period < 3 years) and medium-term (G2, 3 years ≤ follow-up period < 6 years) groups showed no significant difference in PCO value between hydrophobic acrylic and silicone IOLs (G1, [SMD], −0.15; 95% CI, −0.61 to −0.30; *P*=0.51; G2, [SMD], 0.08; 95% CI, −0.22 to 0.39; *P*=0.60). However, in the long term (G3, follow-up period ≥6 years), hydrophobic acrylic IOLs were associated with relatively higher PCO values than silicone IOLs, showing a statistically significant difference (G3 [SMD], −0.59; 95% CI, −0.90 to −0.28; *P*=0.001, Figure 4).

#### 3.4.2. Nd : YAG Capsulotomy Rate

**Figure 5:**
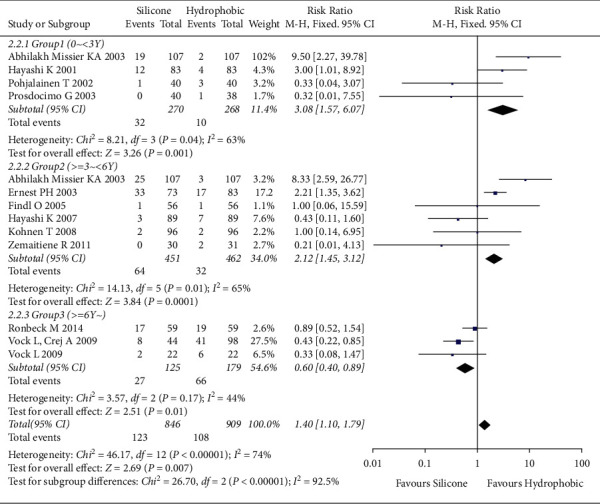
Comparison of subgroup effects on Nd : YAG capsulotomy rates between hydrophobic acrylic and silicone IOLs. In the long-term group (G3, follow-up period ≥6 years), hydrophobic acrylic IOLs were associated with significantly higher Nd : YAG capsulotomy rates than silicone IOLs. Chi^2^ = chi-square statistic; CI = confidence interval; df = degrees of freedom; *I*^2^ = I-squared, heterogeneity statistic; IOL = intraocular lens; M-H = Mantel-Haenszel estimate; RR = risk ratio; *Z* = Z-statistic.

The meta-analysis included 12 studies [6–8, 24, 27–29, 31–34, 37] involving 1,541 eyes. The overall effect showed an intermediate degree of heterogeneity (*I*^2^ = 70%). Therefore, the fixed-effects model was used. The overall effect without considering the follow-up period showed no statistically significant difference in the Nd : YAG capsulotomy rate between hydrophobic acrylic and silicone IOLs ([RR], 1.21; 95% CI, 0.9–1.56; *P*=0.14). The forest plot is detailed in Supplementary File 7. Subgroup analysis during the follow-up period revealed an intermediate degree of heterogeneity (*I*^2^ = 74%). Thus, a fixed-effects model was applied. Short-term (G1, 0 years ≤ follow-up period < 3 years) and medium-term (G2, 3 years ≤ follow-up period < 6 years) groups with hydrophobic acrylic IOLs showed lower Nd : YAG capsulotomy rates than those with silicone IOLs (G1, [RR], 3.08; 95% CI, 1.57–6.07; *P*=0.001, G2, [RR], 2.12; 95% CI, 1.45–3.12; *P* < 0.001). However, in the long-term group (G3, follow-up period ≥6 years), hydrophobic acrylic IOLs resulted in higher Nd : YAG capsulotomy rates than silicone IOLs, showing a statistically significant difference between these two IOLs (G3 [RR], 0.60; 95% CI, 0.40–0.89; *P*=0.01, Figure 5).

### 3.5. Complications Immediately following Cataract Surgery and Visual Function

#### 3.5.1. ACO Value

**Figure 6:**
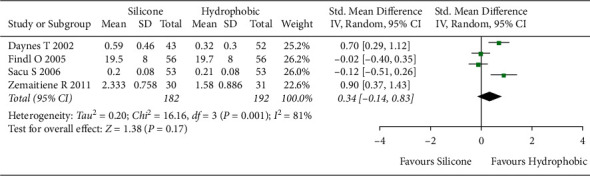
Comparison of ACO values between hydrophobic acrylic and silicone IOLs, showing no statistically significant differences between the two IOLs. Chi^2^ = chi-square statistic; CI = confidence interval; df = degrees of freedom, *I*^2^ = I-squared, heterogeneity statistic; IOL = intraocular lens; IV = inverse variance; SMD = standard mean difference; *Z* = Z-statistic.

The meta-analysis included four studies [26, 27, 35, 37] with 384 eyes to determine the ACO value. The overall effect showed a high heterogeneity (*I*^2^ = 81%). Therefore, the random-effects model was applied. In the forest plot, ACO values of hydrophobic acrylic IOLs were relatively lower than those of silicone IOLs, showing no statistically significant difference ([SMD], 0.34; 95% CI, −0.14 to 0.83; *P*=0.17, Figure 6).

#### 3.5.2. Visual Acuity (BCVA)

**Figure 7:**
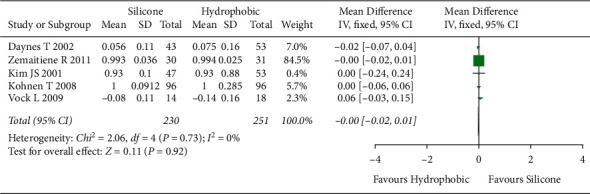
Comparison of visual acuity (BCVA) between hydrophobic acrylic and silicone IOLs, showing no statistically significant differences between the two IOLs. BCVA = best-corrected visual acuity; Chi^2^ = chi-square statistic; CI = confidence interval; df = degrees of freedom; *I*^2^ = I-squared, heterogeneity statistic; IOL = intraocular lens; IV = inverse variance; MD = mean difference; *Z* = Z-statistic.

The meta-analysis included five studies [6, 26, 30, 31, 37] with 481 eyes to determine visual acuity. No statistically significant heterogeneity (*I*^2^ = 0%) was observed between included studies. Therefore, the fixed-effects model was used. The overall effect showed no statistically significant difference in visual acuity between hydrophobic acrylic and silicone IOLs ([MD], −0.00; 95% CI, −0.02 to 0.01; *P*=0.92, Figure 7).

#### 3.5.3. Tilt and Decentration

**Figure 8:**
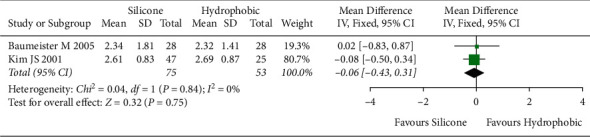
Comparison of tilt between hydrophobic acrylic and silicone IOLs, showing no statistically significant differences between the two IOLs. Chi^2^ = chi-square statistic; CI = confidence interval; df = degrees of freedom; *I*^2^ = I-squared, heterogeneity statistic; IOL = intraocular lens; IV = inverse variance; MD = mean difference; *Z* = Z-statistic.

**Figure 9:**
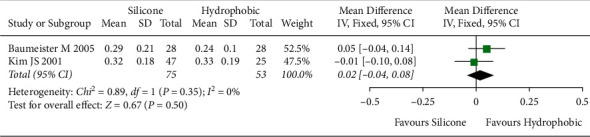
Comparison of decentration between hydrophobic acrylic and silicone IOLs, showing no statistically significant differences between the two IOLs. Chi^2^ = chi-square statistic; CI = confidence interval; df = degrees of freedom; *I*^2^ = I-squared, heterogeneity statistic; IOL = intraocular lens; IV = inverse variance; MD = mean difference; *Z* = Z-statistic.

Two studies [25, 30] with 128 eyes were included to analyze tilt and decentration, respectively. Both outcomes showed no statistically significant heterogeneity between studies (tilt, *I*^2^ = 0%; decentration, *I*^2^ = 0%). Thus, fixed-effects models were applied. Overall effects showed no statistically significant differences between hydrophobic acrylic and silicone IOLs (tilt, [MD], −0.06; 95% CI, −0.43 to 0.31; *P*=0.75, Figure 8; decentration [MD], 0.02; 95% CI, −0.04 to 0.08; *P*=0.50, Figure 9).

### 3.6. Sensitivity Analysis

Sensitivity analysis was performed except for three [27, 31, 36] that did not report SDs. Analysis revealed no significant change in overall results (Supplementary File 8).

### 3.7. Publication Bias

Publication bias was evaluated by visually examining the funnel plot. The funnel plot showed asymmetry in Nd : YAG capsulotomy rate, suggesting some degree of publication bias (Supplementary File 9).

## 4. Discussion

In the present study, systematic review and meta-analysis were conducted to evaluate complications during long-term follow-up and visual function of hydrophobic acrylic IOLs compared with silicone IOLs. The contribution of this study can be summarized as follows. We evaluated the effects of complications and visual function, including long-term clinical studies with follow-up of more than six years after implantation of hydrophobic acrylic or silicone IOLs. We also found that compared with silicone IOLs, hydrophobic acrylic IOLs were better in terms of the degree of PCO [17–19]. However, hydrophobic acrylic IOLs were associated with higher PCO values and Nd : YAG capsulotomy rates over a 6-year follow-up. Therefore, a long-term (≥6 years) use of hydrophobic acrylic IOLs could affect PCO and Nd : YAG capsulotomy more than such use of silicone IOLs.

Subgroup analysis during the follow-up period revealed higher PCO value and Nd : YAG capsulotomy rates in the group carrying long-term (≥6 years) hydrophobic acrylic IOLs compared with those bearing silicone IOLs. This finding was inconsistent with previous studies [17–19] reporting a lower PCO value with hydrophobic acrylic IOLs than that with silicone IOLs. The barrier effect on PCO is generated by a stably formed capsule bending inhibiting the movement of lens epithelial cells (LECs) to the posterior capsule, which is primarily superior in sharp-edge IOLs [38, 39]. However, if the continuous proliferation of LECs is delayed over a specific duration, a Soemmering's Ring is formed, which abrades the barrier effect of the sharp edge [39]. The hydrophobic acrylic IOLs in this study all had sharp edges. In contrast, silicone IOLs partially exhibited round edges. Nonetheless, compared with silicone IOLs, hydrophobic acrylic IOLs exhibited higher PCO values and Nd : YAG capsulotomy rates, implying that the barrier effect of sharp-edge hydrophobic acrylic IOL was lost due to a long-term (≥6 years) use. Thus, from a long-term perspective, it can be interpreted that the properties of the material itself had a greater impact on the PCO than the effects of the edge design. Compared with hydrophobic acrylic, silicone can mediate the adhesion between IOL and capsule by combining collagen IV and vitronectin attachment proteins [40]. Silicone can also resist the formation of Soemmering's Ring [39]. Therefore, it could help prevent PCO longer during the long-term use than hydrophobic acrylic.

The PCO value did not vary significantly between hydrophobic acrylic and silicone IOLs in the short-term (G1) or the medium-term (G2) follow-up. However, the incidence of Nd : YAG capsulotomy rate was lower in the case of hydrophobic acrylic IOLs during short-term (G1) and medium-term (G2) follow-ups. Although Nd : YAG capsulotomy is the only treatment for PCO, the PCO value and the Nd : YAG capsulotomy rate did not show consistency, which was contrary to other studies [7, 41]. This result might be attributed to differences in reaching Nd : YAG capsulotomy diagnosis depending on the degree of PCO. According to Ling et al. [41], the diagnosis of PCO prior to performing Nd : YAG capsulotomy is not always established. It may vary depending on the assessment. Clinical studies analyzed in this meta-analysis used a variety of evaluation systems, including subjective methods for evaluating PCO levels. Unfortunately, no standardized method is currently available to evaluate the PCO value before Nd : YAG capsulotomy in clinical practice [41]. Therefore, advanced methods of PCO standardization and clinical trials with subsequent Nd : YAG capsulotomy are needed.

There was no significant difference in ACO value between hydrophobic acrylic and silicone IOLs. Hydrophobic acrylic IOLs had relatively lower ACO values than silicone IOLs ([SMD] = 0.34), indicating an intermediate effect size (0.2 ≤ [SMD] < 0.5) [42]. This might be due to a more pronounced effect of similar properties between haptic materials of the two IOLs on ACO than optic materials of the IOLs. Silicone IOLs included in our ACO analysis all had three pieces made of polyvinylidene fluoride (PVDF) or PMMA haptics. Loop memory in PVDF has properties similar to PMMA haptic of hydrophobic acrylic IOLs [43]. However, a high heterogeneity (*I*^2^ = 81%) between ACO studies included in the meta-analysis was found. This interpretation is marginal due to the small number of studies. No further analysis of heterogeneity has been made. However, the high heterogeneity might be attributed to a combination of factors and scales that affect ACO.

Comparing the effect size of hydrophobic acrylic and silicone IOLs in terms of visual function after cataract surgery, visual acuity was statistically similar between the two groups. Previous meta-analyses [11, 44, 45] comparing typical IOL materials (PMMA, silicone, and acrylic) have revealed no significant differences in visual acuity. Our study results are consistent with these prior studies, suggesting the absence of a significant effect on the visual acuity of these two IOL materials. There were no significant differences in tilt or decentration between hydrophobic acrylic and silicone IOL materials either. Forward and backward movement of IOL due to tilt and decentration can affect refraction and aberration of eyes [25]. This effect depends on the spherical degree of IOLs, which has recently been complemented by the emergence of aspherical IOLs [46]. All hydrophobic acrylic and silicone IOLs included in this study were spherical, suggesting no difference in optical performance [25, 47].

This meta-analysis has some limitations. Since most clinical studies related to IOL materials mainly reported results of PCO and Nd : YAG capsulotomy, a subgroup analysis was feasible only for PCO value and Nd : YAG capsulotomy rate during the follow-up period. Therefore, an adequate number of clinical trials related to visual function and complications other than PCO are needed in the future. Furthermore, results of ACO suggested a high heterogeneity (*I*^2^ = 81%). No further analysis has been made to decrease the heterogeneity. Another subgroup or sensitivity analysis, such as an additional analysis based on edge design, haptic material, optical size, presence of aspheric lens, and surgical technique used [48, 49], will be necessary in the future. Nd : YAG capsulotomy irradiated with low energy laser affects the morphology of IOL. Thus, further meta-analysis studies should be done to determine damage and structure changes of IOL after being hit by the laser [50].

## 5. Conclusion

Hydrophobic acrylic IOLs are associated with higher PCO values and Nd : YAG capsulotomy rates than silicone IOLs when they are used for a long term (more than 6 years). However, both hydrophobic acrylic and silicone IOLs can lead to similar visual functions.

## Figures and Tables

**Table 1:** Characteristics of RCTs or retrospective studies included in the meta-analysis.

Study	Study design	Country	IOL group	Eyes	Age	Follow-up
Abhilakh Missier et al. [24]	Retrospective study	Netherlands	Hydrophobic acrylic	107	74 ± 14	3 years
			Silicone	107		

Baumeister et al. [25]	RCT	Germany	Hydrophobic acrylic	28	74 ± 7	1 week
			Silicone	28		6, 12 months

Daynes et al. [26]	Retrospective study	USA	Hydrophobic acrylic	60	70	3 years
			Silicone	51	77	

Findl et al. [27]	RCT	Austria	Hydrophobic acrylic	53	78 ± 4	1 year
			Silicone	53		3 years

Hayashi et al. [28]	RCT	Japan	Hydrophobic acrylic	96	68.8 ± 10.5	1 week
			Silicone	83	71.0 ± 8.9	3, 6, 12, 18 months 2 years

Hayashi et al. [29]	RCT	Japan	Hydrophobic acrylic	100	71.4 ± 6.5	3 years
			Silicone	100		

Kim et al. [30]	RCT	Korea	Hydrophobic acrylic	25	63.7 ± 9.2	1, 3, 6 months
			Silicone	47	61.3 ± 10.4	

Kohnen et al. [31]	RCT	Germany	Hydrophobic acrylic	60	73.9	3 years
			Silicone	60		

Ernest et al. [32]	RCT	USA	Hydrophobic acrylic	83	74	3 years
			Silicone	73		

Pohjalainen et al. [33]	RCT	Finland	Hydrophobic acrylic	40	67.1 ± 14.1	2.4 years
			Silicone	40	67.2 ± 13.9	

Prosdocimo et al. [34]	RCT	Italy	Hydrophobic acrylic	38	71	18 months
			Silicone	40		

Rønbeck et al. [7]	RCT	Sweden	Hydrophobic acrylic	62	73.1	12 years
			Silicone	64		

Sacu et al. [35]	RCT	Austria	Hydrophobic acrylic	53	78 ± 4	1 year
			Silicone	53		

Vock et al. [6]	Retrospective study	Austria	Hydrophobic acrylic	98	M: 66.4 ± 10.1	10 years
					F: 68.1 ± 10.1	
			Silicone	44	M: 65.6 ± 7.8	
					F: 69.8 ± 6.5	

Vock, Crnej et al. [8]	RCT	Austria	Hydrophobic acrylic	53	75 ± 9	6 years
			Silicone	53	75	

Wejde et al. [36]	RCT	Sweden	Hydrophobic acrylic	59	75	3 years
			Silicone	60	73	

Zemaitiene et al. [37]	RCT	Lithuania	Hydrophobic acrylic	34	67.6 ± 7.7	3 years
			Silicone	30		

All included patients had age-related cataract; age is reported in years. F = female; IOL = intraocular lens; M = male; RCT = randomized controlled trial.

## Data Availability

All data are included within this article and its supplementary files.

## Supplementary Materials

Supplementary Materials
*Supplementary File 1.* PRISMA 2020 checklist. *Supplementary File 2.* Details of the search strategy. *Supplementary File 3.* Details of excluded studies and reasons. *Supplementary File 4.* Characteristics of IOLs included studies. *Supplementary File 5.* Risk of bias assessment of randomized controlled trials. *Supplementary File 6.* Risk of bias assessment of nonrandomized controlled trials. *Supplementary File 7.* Forest plots of the overall effect of PCO value and Nd : YAG capsulotomy rate. *Supplementary File 8.* Sensitivity analysis except the studies missing SDs. *Supplementary File 9.* Funnel plots of publication bias.
