# Supplementary material for: Posterior Capsule Opacification after Cataract Surgery via Implantation with Hydrophobic Acrylic Lens Compared with Silicone Intraocular Lens: A Systematic Review and Meta-Analysis
Source: J Ophthalmol. 2022 Feb 25;2022:3570399. doi: 10.1155/2022/3570399 (PMC8896947; doi:10.1155/2022/3570399)

Supplementary File 1. PRISMA 2020 Checklist

Section and Topic	Item #	Checklist item	Location where item is reported (page#)
**TITLE**			
Title	1	Identify the report as a systematic review.	1
**ABSTRACT**			
Abstract	2	See the PRISMA 2020 for Abstracts checklist.	1
**INTRODUCTION**			
Rationale	3	Describe the rationale for the review in the context of existing knowledge.	2
Objectives	4	Provide an explicit statement of the objective(s) or question(s) the review addresses.	2
**METHODS**			
Eligibility criteria	5	Specify the inclusion and exclusion criteria for the review and how studies were grouped for the syntheses.	3
Information sources	6	Specify all databases, registers, websites, organisations, reference lists and other sources searched or consulted to identify studies. Specify the date when each source was last searched or consulted.	2
Search strategy	7	Present the full search strategies for all databases, registers and websites, including any filters and limits used.	Table S2 (Supplementary file 2)
Selection process	8	Specify the methods used to decide whether a study met the inclusion criteria of the review, including how many reviewers screened each record and each report retrieved, whether they worked independently, and if applicable, details of automation tools used in the process.	3
Data collection process	9	Specify the methods used to collect data from reports, including how many reviewers collected data from each report, whether they worked independently, any processes for obtaining or confirming data from study investigators, and if applicable, details of automation tools used in the process.	3
Data items	10a	List and define all outcomes for which data were sought. Specify whether all results that were compatible with each outcome domain in each study were sought (e.g. for all measures, time points, analyses), and if not, the methods used to decide which results to collect.	3
	10b	List and define all other variables for which data were sought (e.g. participant and intervention characteristics, funding sources). Describe any assumptions made about any missing or unclear information.	3
Study risk of bias assessment	11	Specify the methods used to assess risk of bias in the included studies, including details of the tool(s) used, how many reviewers assessed each study and whether they worked independently, and if applicable, details of automation tools used in the process.	3
Effect measures	12	Specify for each outcome the effect measure(s) (e.g. risk ratio, mean difference) used in the synthesis or presentation of results.	3, 4
Synthesis methods	13a	Describe the processes used to decide which studies were eligible for each synthesis (e.g. tabulating the study intervention characteristics and comparing against the planned groups for each synthesis (item #5)).	N/A
	13b	Describe any methods required to prepare the data for presentation or synthesis, such as handling of missing summary statistics, or data conversions.	3
	13c	Describe any methods used to tabulate or visually display results of individual studies and syntheses.	N/A
	13d	Describe any methods used to synthesize results and provide a rationale for the choice(s). If meta-analysis was performed, describe the model(s), method(s) to identify the presence and extent of statistical heterogeneity, and software package(s) used.	4
	13e	Describe any methods used to explore possible causes of heterogeneity among study results (e.g. subgroup analysis, meta-regression).	4

Section and Topic	Item #	Checklist item	Location where item is reported (page#)
	13f	Describe any sensitivity analyses conducted to assess robustness of the synthesized results.	3
Reporting bias assessment	14	Describe any methods used to assess risk of bias due to missing results in a synthesis (arising from reporting biases).	3
Certainty assessment	15	Describe any methods used to assess certainty (or confidence) in the body of evidence for an outcome.	N/A
**RESULTS**			
Study selection	16a	Describe the results of the search and selection process, from the number of records identified in the search to the number of studies included in the review, ideally using a flow diagram.	4
	16b	Cite studies that might appear to meet the inclusion criteria, but which were excluded, and explain why they were excluded.	Table S3 (Supplementary file 3)
Study characteristics	17	Cite each included study and present its characteristics.	5
Risk of bias in studies	18	Present assessments of risk of bias for each included study.	6-7
Results of individual studies	19	For all outcomes, present, for each study: (a) summary statistics for each group (where appropriate) and (b) an effect estimate and its precision (e.g. confidence/credible interval), ideally using structured tables or plots.	Figure 4-9
Results of syntheses	20a	For each synthesis, briefly summarise the characteristics and risk of bias among contributing studies.	Table S5, S6 (Supplementary file 5, 6)
	20b	Present results of all statistical syntheses conducted. If meta-analysis was done, present for each the summary estimate and its precision (e.g. confidence/credible interval) and measures of statistical heterogeneity. If comparing groups, describe the direction of the effect.	7-10
	20c	Present results of all investigations of possible causes of heterogeneity among study results.	12
	20d	Present results of all sensitivity analyses conducted to assess the robustness of the synthesized results.	10
Reporting biases	21	Present assessments of risk of bias due to missing results (arising from reporting biases) for each synthesis assessed.	11
Certainty of evidence	22	Present assessments of certainty (or confidence) in the body of evidence for each outcome assessed.	N/A
**DISCUSSION**			
Discussion	23a	Provide a general interpretation of the results in the context of other evidence.	11-12
	23b	Discuss any limitations of the evidence included in the review.	12
	23c	Discuss any limitations of the review processes used.	12
	23d	Discuss implications of the results for practice, policy, and future research.	12-13
**OTHER INFORMATION**			
Registration and	24a	Provide registration information for the review, including register name and registration number, or state that the review was not registered.	N/A

Section and Topic	Item #	Checklist item	Location where item is reported (page#)
protocol	24b	Indicate where the review protocol can be accessed, or state that a protocol was not prepared.	2
	24c	Describe and explain any amendments to information provided at registration or in the protocol.	N/A
Support	25	Describe sources of financial or non-financial support for the review, and the role of the funders or sponsors in the review.	13
Competing interests	26	Declare any competing interests of review authors.	13
Availability of data, code and other materials	27	Report which of the following are publicly available and where they can be found: template data collection forms; data extracted from included studies; data used for all analyses; analytic code; any other materials used in the review.	Specified the available sources

From: Page MJ, McKenzie JE, Bossuyt PM, Boutron I, Hoffmann TC, Mulrow CD, et al. The PRISMA 2020 statement: an updated guideline for reporting systematic reviews. BMJ 2021;372:n71. doi: 10.1136/bmj.n71

For more information, visit: http://www.prisma-statement.org/

Supplementary File 2. Search strategy

Database	Search terms
**PubMed**	Article types: Clinical Study, Clinical Trial, Comparative Study, Randomized Controlled Trial and 2000-01-01~2021-03-01<br>((cataract*[MeSH Terms]) OR (cataract extraction*[MeSH Terms]) OR (capsule opacification*[MeSH Terms])) AND ((silicone) OR (hydrophobic acrylic) AND ((IOL) OR (intraocular lens[MeSH Terms]) OR (intraocular lens implantation*[MeSH Terms]) OR (lenses, intraocular[MeSH Terms]) OR (lens implantation, intraocular[MeSH Terms]))) NOT ((pediatric cataract*) OR (children))
**Embase**	Article types: Controlled clinical trial, Randomized controlled trial and 2000~2021<br>('cataract*'/exp OR 'cataract extraction*'/exp OR 'cataract operation*'/exp OR 'cataract surgery'/exp) AND (('intraocular lens*'/exp OR 'IOL'/exp OR 'lens implantation*'/exp OR 'lens implant*'/exp) AND ('silicone'/exp OR 'Hydrophobic acrylic'/exp))
**Cochrane library**	Article types: Trials and 2000-01-01~2021-03-01<br>("intraocular lens"):ti AND ("silicone"):ti OR (hydrophobic acrylic):ti NOT ("pediatric"):ti AND ("cataract"):ti,ab,kw (Word variations have been searched)

Supplementary File 3. Excluded studies and reasons

Authors	Title	Reason for exclusion
Abela-Formanek C et al. 2002	Inflammation after implantation of hydrophilic acrylic, hydrophobic acrylic, or silicone intraocular lenses in eyes with cataract and uveitis: comparison to a control group	Publication type not of interest
Abela-Formanek C et al. 2002	Uveal and capsular biocompatibility of hydrophilic acrylic, hydrophobic acrylic, and silicone intraocular lenses	Insufficient data
Abela-Formanek C et al. 2002	Results of hydrophilic acrylic, hydrophobic acrylic, and silicone intraocular lenses in uveitic eyes with cataract: comparison to a control group	Insufficient data
Auffarth GU et al. 2003	Quantification of posterior capsule opacification with round and sharp edge intraocular lenses	Publication type not of interest
Auffarth GU et al. 2003	Comparison of Nd : YAG capsulotomy rates following phacoemulsification with implantation of PMMA, silicone, or acrylic intra-ocular lenses in four European countries	diabetes requiring medical control
Beltrame G et al. 2002	Posterior capsule opacification and Nd:YAG capsulotomy rates after implantation of silicone, hydrogel and soft acrylic intraocular lenses: a two-year follow-up study	Only abstract
Ding Y et al. 2009	Quantification of posterior capsular opacification after cataract surgery	Only abstract
Elgohary MA et al. 2006	Optical coherence tomography of intraocular lens implants and their relationship to the posterior capsule: a pilot study comparing a hydrophobic acrylic to a plate-haptic silicone type	Publication type not of interest
Georgopoulos M et al. 2003	Influence of intraocular lens material on regeneratory posterior capsule opacification after neodymium:YAG laser capsulotomy	Insufficient data
Halpern MT et al. 2002	Relationship of AcrySof acrylic and PhacoFlex silicone intraocular lenses to visual acuity and posterior capsule opacification	Insufficient data
Hütz WW et al. 2012	Comparison of visual performance of silicone and acrylic multifocal IOLs utilizing the same diffractive design	Multifocal IOLs
Hwang IP et al. 2001	Patient satisfaction after uneventful cataract surgery with implantation of a silicone or acrylic foldable intraocular lens. Comparative study	No wanted outcome
Jung CK et al. 2000	Decentration and tilt: silicone multifocal versus acrylic soft intraocular lenses	Multifocal IOLs
Kremmer S et al. 2003	Influence of cataract surgery with implantation of different intraocular lenses on scanning laser tomography and polarimetry	Publication type not of interest
Kremmer S et al. 2003	Effect of AcrySof versus silicone or polymethyl methacrylate intraocular lens on posterior capsule opacification	Publication type not of interest
Ober MD et al. 2000	Posterior capsular opacification in phacotrabeculectomy : a long-term comparative study of silicone versus acrylic intraocular lens	Patients not of interest
Papaliodis GN et al. 2002	Intraocular lens tolerance in surgery for cataracta complicata: assessment of four implant materials	Insufficient data
Ram J et al. 2001	Neodymium:YAG capsulotomy rates following phacoemulsification with implantation of PMMA, silicone, and acrylic intraocular lenses	Only abstract
Schrecker J et al. 2014	Silicone-diffractive versus acrylic-refractive supplementary iols: visual performance and manual handling	Multifocal IOLs

Supplementary File 4. Characteristics of intraocular lenses included studies

Study	IOL group	Model	Piece number	Haptic material	Edge design	PCO/ACO evaluation system
Abhilakh Missier KA et al. 2003 [26]	Hydrophobic acrylic	AcrySof MA30BA/MA60BM	3	PMMA	sharp	EPCO
	Silicone	Staar AA4203VF	1	plate-haptic	N/A	
Baumeister M et al. 2005 [27]	Hydrophobic acrylic	AcrySof MA60	3	PMMA	sharp	N/A
	Silicone	CeeOn Edge 911A	3	PVDF	sharp	
Daynes T et al. 2002 [28]	Hydrophobic acrylic	AcrySof MA60/MA30	3	PMMA	sharp	EPCO
	Silicone	SI-40NB	3	PMMA	round	
Findl O et al. 2005 [29]	Hydrophobic acrylic	AcrySof MA60BM	3	PMMA	sharp	AQUA
	Silicone	CeeOn Edge 911A	3	PVDF	sharp	
Hayashi K et al. 2001 [30]	Hydrophobic acrylic	AcrySof MA60BM	3	PMMA	sharp	Scheimpflug
	Silicone	SI-30NB	3	polypropylene	round	
Hayashi K et al. 2007 [31]	Hydrophobic acrylic	AR40e	3	PMMA	sharp	Scheimpflug
	Silicone	ClariFlex	3	PMMA	sharp	
Kim JS et al. 2001 [32]	Hydrophobic acrylic	AcrySof MA60BM	3	PMMA	sharp	N/A
	Silicone	SI-30NB	3	polypropylene	round	
Kohnen T et al. 2008 [33]	Hydrophobic acrylic	AcrySof MA60BM	3	PMMA	sharp	EPCO
	Silicone	CeeOn Edge 911A	3	PVDF	sharp	
Ernest PH et al. 2003 [34]	Hydrophobic acrylic	AcrySof MA30BA	3	PMMA	sharp	N/A
	Silicone	SI-40NB	3	PMMA	round	
Pohjalainen T et al. 2002 [35]	Hydrophobic acrylic	AcrySof MA60BM	3	PMMA	sharp	N/A
	Silicone	SI-30NB	3	polypropylene	round	
Prosdocimo G et al. 2003 [36]	Hydrophobic acrylic	AcrySof	N/A	PMMA	sharp	AQUA
	Silicone	CeeOn Edge 911A	3	PVDF	sharp	
Ronbeck M et al. 2014 [8]	Hydrophobic acrylic	Acrysof MA60BM	3	PMMA	sharp	POCOman
	Silicone	SI-40NB	3	PMMA	round	
Sacu S et al. 2006 [37]	Hydrophobic acrylic	Acrysof MA60BM	3	PMMA	sharp	Adobe photoshop
	Silicone	CeeOn Edge 911A	3	PVDF	sharp	
Vock L et al. 2009 [7]	Hydrophobic acrylic	AcrySof MA60BM	3	PMMA	sharp	AQUA
	Silicone	SI-30NB/SI-40NB	3	Polypropylene/PMMA	round	
Vock L, Crnej A et al. 2009 [9]	Hydrophobic acrylic	AcrySof MA60BM	3	PMMA	sharp	AQUA
	Silicone	CeeOn Edge 911A	3	PVDF	sharp	
Wejde G et al. 2004 [38]	Hydrophobic acrylic	AcrySof MA60BM	3	PMMA	sharp	EPCO
	Silicone	SI-40NB	3	PMMA	round	
Zemaitiene R et al. 2011 [39]	Hydrophobic acrylic	AcrySof MA30BA	3	PMMA	sharp	EPCO
	Silicone	CeeOn Edge 911A	3	PVDF	sharp	

ACO = anterior capsule opacification; IOL = intraocular lens; PMMA = polymethyl methacrylate; PVDF = polyvinylidene fluoride; PCO = posterior capsule opacification.

Supplementary File 5. Risk of bias assessment of randomized controlled trials

Baumeister M 2005 [27]

Bias	Judgement	Support for judgement
Random sequence generation (selection bias)	Unclear	"Patient randomization for the entire multicenter study were performed by the institute for Medical Statistics, Computer Science and Documentation of the Friedrich Schiller University, Jena, on behalf of Pharmacia Co."
Allocation concealment (selection bias)	Low	The IOL to be implanted in the first eye was assigned according to a randomization scheme.
Blinding of participants and personnel (performance bias)	High	"Both examiners were informed about the study and the different shapes of the IOLs. Thus, blinding of the examiners was not possible"
Blinding of outcome assessment (detection bias)	Unclear	Not reported
Incomplete outcome data (attrition bias)	Low	A chart of participant flow was provided, and there were no missing values.
Selective reporting (reporting bias)	Low	Approved protocol<br>Results for predetermined outcomes were reported.
Other bias	Low	Not likely

Ernest PH 2003 [34]

Bias	Judgement	Support for judgement
Random sequence generation (selection bias)	Unclear	"Patients received 1 lens type in 1 eye and the other in the fellow eye and were randomized as to which lens was implanted first and in which eye" No further description of randomization provided.
Allocation concealment (selection bias)	Unclear	Not reported.
Blinding of participants and personnel (performance bias)	Unclear	Not reported
Blinding of outcome assessment (detection bias)	Unclear	Not reported
Incomplete outcome data (attrition bias)	Low	No dropouts
Selective reporting (reporting bias)	Low	Results for predetermined outcomes were reported.
Other bias	High	"Financial support by Alcon laboratories, Inc., Fort Worth, Texas, USA. The author became a paid consultant of Alcon laboratories, Inc., approximately 3 years after the initiation of this study"

Findl O 2005 [29]

Bias	Judgement	Support for judgement
Random sequence generation (selection bias)	Low	"A randomization schedule of 60 allocations was supplied from a computer-derived list of random numbers"
Allocation concealment (selection bias)	Low	Each patient was allocated a unique trial number.
Blinding of participants and personnel (performance bias)	Low	Patient- and examiner-masked
Blinding of outcome assessment (detection bias)	High	"The examiner who performed the slit-lamp examination obviously could not be masked any longer"
Incomplete outcome data (attrition bias)	Low	A chart of participant flow was provided.
Selective reporting (reporting bias)	Low	A supporting protocol existed<br>Results for predetermined outcomes were reported.
Other bias	Low	Not likely

Hayashi K 2001 [30]

Bias	Judgement	Support for judgement
Random sequence generation (selection bias)	Low	Initially randomized into 3 groups based on IOL type. All enrolled eyes were randomly allocated using the sealed-envelope method.
Allocation concealment (selection bias)	Low	All enrolled eyes were randomly allocated using the sealed-envelope method.
Blinding of participants and personnel (performance bias)	Low	Patients, examiners, and surgeons were masked.
Blinding of outcome assessment (detection bias)	Low	Patients, examiners, and surgeons were masked.
Incomplete outcome data (attrition bias)	Low	"Of the 300 eyes, 10 in the PMMA IOL group, 17 in the silicone IOL group, and 4 in the acrylic IOL group were lost to follow-up. Thus, 269 eyes completed a 2 year follow-up and were available for analysis"
Selective reporting (reporting bias)	Low	"The study protocol was approved by the Institutional Review Board, and informed consent was obtained from each patient"
Other bias	Low	Not likely

Hayashi K 2007 [31]

Bias	Judgement	Support for judgement
Random sequence generation (selection bias)	Low	"The controller of this clinical trial generated a randomization code with equal numbers using random number tables, and, to ensure allocation concealment, the assignment schedule was kept concealed until all data were collected"
Allocation concealment (selection bias)	Low	All enrolled patients were randomly assigned the day before surgery to one of two groups.
Blinding of participants and personnel (performance bias)	Low	All patients and examiners were masked as to randomization.
Blinding of outcome assessment (detection bias)	Low	"The operating room personnel who allocated the IOLs to the patients were unaware of the purpose of this study. The examiners were also unaware of the type of IOL used because the two IOLs are the same in appearance. Furthermore, because the controller of this clinical trial assignment schedule was kept concealed until the end of the study, the data analyst, who was the surgeon, did not know the type of IOL used"
Incomplete outcome data (attrition bias)	Low	"Of the 100 patients enrolled, nine were lost to follow-up during the 36-month period: one patient died and two were hospitalized for an unrelated cause, one moved from the area, and five did not appear for reexamination because of an illness or scheduling conflict. In addition, in two patients the Scheimpflug image obtained was difficult to analyze. Therefore, 89 patients (89%) remained for analysis"
Selective reporting (reporting bias)	Low	Protocol approved<br>Results for predetermined outcomes were reported.
Other bias	Low	Not likely

Kim JS 2001 [32]

Bias	Judgement	Support for judgement
Random sequence generation (selection bias)	Low	Using the sealed envelope method, the eyes were stratified randomly into 3 groups based on the IOL type.
Allocation concealment (selection bias)	Low	Using the sealed envelope method
Blinding of participants and personnel (performance bias)	Unclear	Not reported
Blinding of outcome assessment (detection bias)	Unclear	Not reported
Incomplete outcome data (attrition bias)	Low	"Twenty-one patients (25 eyes) did not complete the follow-up, and these eyes were excluded, leaving 137 eyes for analysis"
Selective reporting (reporting bias)	Low	Results for predetermined outcomes were reported.
Other bias	Unclear	Uncertainty existed

Kohnen T 2008 [33]

Bias	Judgement	Support for judgement
Random sequence generation (selection bias)	Low	"The IOL type for the first operated eye was randomly assigned according to a code generated from a random-number table with blocking and stratification by center"
Allocation concealment (selection bias)	Low	Randomly assigned (open-label)
Blinding of participants and personnel (performance bias)	Unclear	Not reported
Blinding of outcome assessment (detection bias)	Unclear	Not reported
Incomplete outcome data (attrition bias)	Low	"Of the 288 randomized patients, 41 (14%) had to be excluded from analyses due to various reasons: severe complications during surgery (10), no surgery or adverse events before second surgery (7), refused further participation (12), and other (12)"
Selective reporting (reporting bias)	Low	Results for predetermined outcomes were reported.
Other bias	Unclear	Uncertainty existed

Pohjalainen T 2002 [35]

Bias	Judgement	Support for judgement
Random sequence generation (selection bias)	Unclear	No further description of randomization was provided.
Allocation concealment (selection bias)	Unclear	Not reported
Blinding of participants and personnel (performance bias)	Unclear	Not reported
Blinding of outcome assessment (detection bias)	Unclear	Not reported
Incomplete outcome data (attrition bias)	Low	No missing values
Selective reporting (reporting bias)	Low	Results for predetermined outcomes were reported.
Other bias	Low	Not likely

Prosdocimo G 2003 [36]

Bias	Judgement	Support for judgement
Random sequence generation (selection bias)	Unclear	"In an open clinical study, 78 cataract patients were randomly selected to have implantation of a silicone CeeOn Edge (Pharmacia) or acrylate AcrySof (Alcon) IOL after phacoemulsification cataract surgery" No further description of randomization provided.
Allocation concealment (selection bias)	Unclear	No further description of randomization was provided.
Blinding of participants and personnel (performance bias)	Unclear	Not reported
Blinding of outcome assessment (detection bias)	Unclear	Not reported
Incomplete outcome data (attrition bias)	Low	Not reported of SD but IQR reported
Selective reporting (reporting bias)	Low	All patients provided informed consent, and the data were collected in accordance with the International Standard Organization protocol for IOL studies.
Other bias	Low	Not likely

Rønbeck M 2014 [8]

Bias	Judgement	Support for judgement
Random sequence generation (selection bias)	Low	A randomization protocol was generated using computer software.
Allocation concealment (selection bias)	Low	The patients were assigned a study number that corresponded to 1 of the 3 IOLs.
Blinding of participants and personnel (performance bias)	Unclear	Not reported
Blinding of outcome assessment (detection bias)	Unclear	Not reported
Incomplete outcome data (attrition bias)	Low	"Postoperatively, at 11.3 to 13.4 years (mean 12.3 years), 74 (39 women, 35 men) of the initial 180 patients were lost to follow-up; 52 patients died, 3 patients moved, 2 patients had dementia, 2 patients had an unknown illness, and 13 patients did not show up for unknown reasons. In addition, 1 patient was lost to follow-up because of aphasia and paralysis after a stroke and 1 patient was excluded because of intraoperative posterior capsule rupture… The statistical analysis of the median Nd:YAG survival time and the mean Nd:YAG overall survival included 179 patients; 1 patient in the silicone IOL group with intraoperative capsule rupture was excluded."
Selective reporting (reporting bias)	Low	Results for predetermined outcomes were reported.
Other bias	Low	Not likely

Sacu S 2006 [37]

Bias	Judgement	Support for judgement
Random sequence generation (selection bias)	Unclear	"Posterior capsule opacification data from these patients have been published previously. The patients were recruited from a continuous cohort" No further description of randomization provided.
Allocation concealment (selection bias)	Unclear	"The IOL type for the first-operated eye of each patient was assigned randomly before surgery" No mention how to be assigned randomly.
Blinding of participants and personnel (performance bias)	Low	Double-blind
Blinding of outcome assessment (detection bias)	Unclear	Not reported
Incomplete outcome data (attrition bias)	Low	"Of the 52 patients who were included in the study, 43 patients were available 1 year after surgery. Nine patients were not available for follow-up examination (one patient died before the 1-year follow-up examination; three patients were excluded after the operation because they were not operated bilaterally, and five patients could not be reached). One patient in group 1 and three patients group 2 were excluded because the pupil dilation did not exceed the size of the capsulorrhexis edge, so 80 eyes of 40 patients were evaluated in each group"
Selective reporting (reporting bias)	Unclear	"The Ethics Committee of the Medical University of Vienna approved the protocol. Patients gave informed consent before inclusion into the study"
Other bias	Low	Not likely

Vock L, Crnej A 2009 [9]

Bias	Judgement	Support for judgement
Random sequence generation (selection bias)	Low	Randomized by using numbers from a computer-generated list of random numbers.
Allocation concealment (selection bias)	Low	“The first eye to be operated in each patient was randomly assigned to receive the silicone IOL (CeeOn Edge 911A) or the acrylic IOL (AcrySof MA60BM)”
Blinding of participants and personnel (performance bias)	Low	Patient- and examiner-masked
Blinding of outcome assessment (detection bias)	Unclear	Not reported
Incomplete outcome data (attrition bias)	Low	“Six other patients passed away during the follow-up period and one became a nursing case. In the other cases, the patients could not be traced and contacted anymore” A chart of participant flow was provided.
Selective reporting (reporting bias)	Unclear	Uncertainty existed
Other bias	Low	Not likely

Wejde G 2004 [38]

Bias	Judgement	Support for judgement
Random sequence generation (selection bias)	Unclear	“The patients were randomized to implantation with either a silicone intraocular lens (IOL) (SI40NB, Allergan) or an AcrySof IOL (MA60BM, Alcon)” No further description of randomization was provided.
Allocation concealment (selection bias)	Unclear	Not reported
Blinding of participants and personnel (performance bias)	Unclear	Not reported
Blinding of outcome assessment (detection bias)	Unclear	Not reported
Incomplete outcome data (attrition bias)	Low	“Twenty-seven patients were lost to follow-up because they were not available for examination or were excluded because the images did not visualize the entire anterior capsulorhexis margin”
Selective reporting (reporting bias)	Low	Results for predetermined outcomes were reported.
Other bias	Low	Not likely

Zemaitienė R 2011 [39]

Bias	Judgement	Support for judgement
Random sequence generation (selection bias)	Unclear	“After the patients provided informed consent, they were randomly assigned to receive a 3-piece AcrySof MA3OBA hydrophobic acrylic IOL or 1-piece AcrySof SA3OAL hydrophobic acrylic IOL or 3-piece CeeOn 911A silicone IOL” No further description of randomization was provided.
Allocation concealment (selection bias)	Unclear	Not reported
Blinding of participants and personnel (performance bias)	High	Non-blinded
Blinding of outcome assessment (detection bias)	High	Non-blinded
Incomplete outcome data (attrition bias)	Low	“Seven patients were known to have died, and 6 patients were too ill or frail to attend. It was not possible to contact 2 patients. Three patients refused to participate in the study”
Selective reporting (reporting bias)	Low	Results for predetermined outcomes were reported.
Other bias	Low	Not likely

Supplementary File 6. Risk of bias assessment of non-randomized controlled trials

Abhilakh Missier KA 2003 [26]

Bias	Judgement	Support for judgement
Bias due to confounding	Low	No prognostic variables (factors that predict the outcome of interest) and no changed IOLs
Bias due to selection of participants	Low	"In each patient, 1 eye was randomly selected to receive an MA30BA (n=77) or MA60BM (n=30) AcrySof acrylate IOL and the other eye, an AA4203VF plate-haptic silicone IOL. Randomization was performed using computerized random number generator"
Bias in classification of interventions	Low	Randomization was performed using a computerized random number generator.
Bias due to deviations from intended interventions	Low	No systematic differences between intervention and comparison groups
Bias due to missing data	Low	No missing patients (All patients were examined.)
Bias in measurement of outcomes	Low	All follow-up visits were performed by the same observer (K.A.A.M)
Bias in selection of the reported result	Low	No any suspicious reports

Daynes T 2002 [28]

Bias	Judgement	Support for judgement
Bias due to confounding	Low	No prognostic variables (factors that predict the outcome of interest) and no changed IOLs
Bias due to selection of participants	High	"Patients with at least 3 years of follow-up were reviewed consecutively and retrospectively for evidence of uneventful surgery with no evidence of sight-limiting pathology and at least 20/25 uncorrected visual acuity in the early postoperative period. Patients who met these preliminary criteria were called consecutively and asked to come in for a comprehensive examination. Approximately 60% of patients were contacted; half agreed to come for the examination" Only eligible patients were included (good visual acuity) so there was no examination of all patients (60%).
Bias in classification of interventions	Low	No suspicious bias of classification
Bias due to deviations from intended interventions	Low	No systematic differences between intervention and comparison groups
Bias due to missing data	Low	All of the responded patients were reported but 60%.
Bias in measurement of outcomes	Low	All examinations were carried out in a masked fashion.
Bias in selection of the reported result	Low	No any suspicious reports

Vock L 2009 [7]

Bias	Judgement	Support for judgement
Bias due to confounding	Low	No prognostic variables (factors that predict the outcome of interest) and no changed IOLs
Bias due to selection of participants	Low	"Patients having had cataract surgery and implantation of at least 1 study IOL by the same surgeon between 1994 and 1999 were retrospectively examined. These patients were recruited and invited by letter to have a voluntary eye examination. Of 298 eligible patients, 98 accepted the invitation and 46 were reported to have died; the others did not respond to the invitation for unknown reasons"
Bias in classification of interventions	Low	No suspicious bias of classification
Bias due to deviations from intended interventions	Low	No systematic differences between intervention and comparison groups
Bias due to missing data	Low	Showed with/without imputation of missing values
Bias in measurement of outcomes	Low	Use of the same evaluation software
Bias in selection of the reported result	Low	No any suspicious reports

Supplementary File 7. Forest plots

a. The overall effect of PCO value (Chi^2 = chi-square statistic, CI = confidence interval, df = degrees of freedom, I^2 = I-squared, heterogeneity statistic, IV = inverse variance, SMD = standard mean difference, Z = Z-statistic).

	Silicone			Hydrophobic				Std. Mean Difference
Study or Subgroup	Mean	SD	Total	Mean	SD	Total	Weight	IV, Random, 95% CI
Abhilakh Missier KA 2003	1.178	1.636	107	2.056	1.415	107	11.4%	-0.57 [-0.85, -0.30]
Daynes T 2002	0.42	0.52	43	0.55	0.66	52	10.0%	-0.21 [-0.62, 0.19]
Findl O 2005	1.9	1.6	56	2.2	1.6	56	10.4%	-0.19 [-0.56, 0.19]
Hayashi K 2001	14.1	9.2	83	11.7	7.6	96	11.2%	0.29 [-0.01, 0.58]
Kohnen T 2008	0.0005	0.235	60	0.044	0.289	60	10.5%	-0.16 [-0.52, 0.19]
Prosdocimo G 2003	0.089	0.17	40	0.365	0.47	38	9.4%	-0.78 [-1.24, -0.32]
Vock L, Crnej A 2009	1.4	2.6	44	2.76	2.78	99	10.5%	-0.50 [-0.86, -0.14]
Vock L 2009	2.3	1.4	22	3.8	2	22	7.8%	-0.85 [-1.47, -0.23]
Wejde G 2004	0.223	0.235	47	0.054	0.289	45	9.9%	0.64 [0.22, 1.06]
Zemaitiene R 2011	0.158	0.194	30	0.171	0.208	31	9.0%	-0.06 [-0.57, 0.44]
**Total (95% CI)**			**532**			**606**	**100.0%**	**-0.23 [-0.50, 0.05]**

Heterogeneity: Tau² = 0.16; Chi² = 46.08, df = 9 (P < 0.00001); I² = 80%
Test for overall effect: Z = 1.61 (P = 0.11)

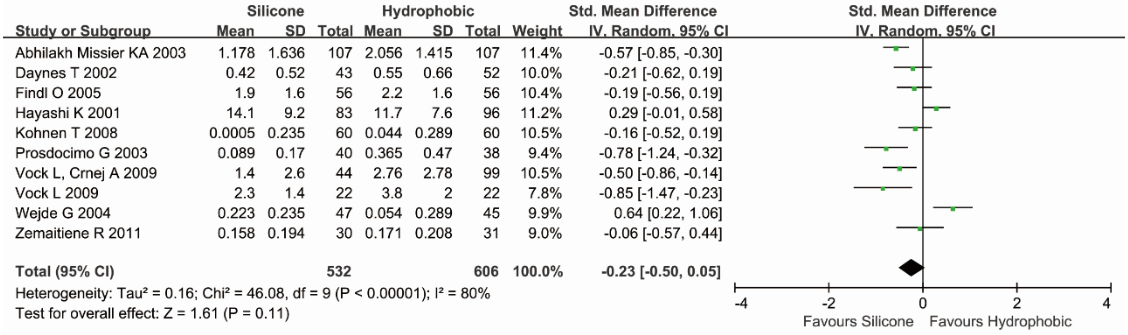

b. The overall effect of Nd:YAG capsulotomy rate (Chi^2 = chi-square statistic, CI = confidence interval, df = degrees of freedom, I^2 = I-squared, heterogeneity statistic, M-H = Mantel-Haenszel estimate, RR = risk ratio, Z = Z-statistic).

	Silicone		Hydrophobic			Risk Ratio
Study or Subgroup	Events	Total	Events	Total	Weight	M-H, Fixed, 95% CI
Abhilakh Missier KA 2003	25	107	3	107	3.3%	8.33 [2.59, 26.77]
Ernest PH 2003	33	74	17	82	17.8%	2.15 [1.31, 3.52]
Findl O 2005	1	56	1	56	1.1%	1.00 [0.06, 15.59]
Hayashi K 2001	12	83	4	83	4.4%	3.00 [1.01, 8.92]
Hayashi K 2007	3	89	7	89	7.7%	0.43 [0.11, 1.60]
Kohnen T 2008	2	96	2	96	2.2%	1.00 [0.14, 6.95]
Pohjalainen T 2002	1	40	3	40	3.3%	0.33 [0.04, 3.07]
Prosdocimo G 2003	0	40	1	38	1.7%	0.32 [0.01, 7.55]
Ronbeck M 2014	17	59	19	59	21.0%	0.89 [0.52, 1.54]
Vock L, Crnej A 2009	8	44	41	98	28.1%	0.43 [0.22, 0.85]
Vock L 2009	2	22	6	22	6.6%	0.33 [0.08, 1.47]
Zemaitiene R 2011	0	30	2	31	2.7%	0.21 [0.01, 4.13]
**Total (95% CI)**		**740**		**801**	**100.0%**	**1.21 [0.94, 1.56]**
Total events	104		106			

Heterogeneity: Chi² = 37.21, df = 11 (P = 0.0001); I² = 70%
Test for overall effect: Z = 1.49 (P = 0.14)

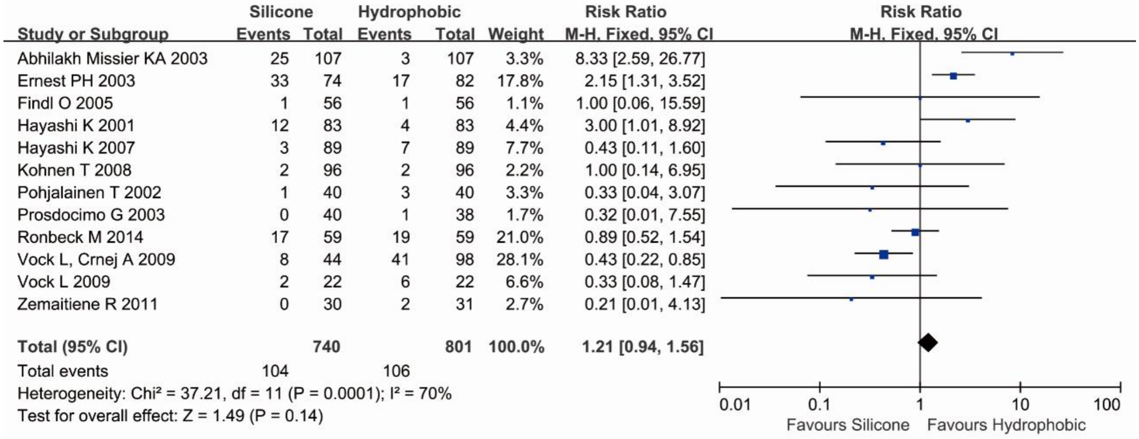

Supplementary File 8. Sensitivity analysis

a. Subgroup analysis of PCO value (Chi2 = chi-square statistic, CI = confidence interval, df = degrees of freedom, I2 = I-squared, heterogeneity statistic, IV = inverse variance, SMD = standard mean difference, Z = Z-statistic).

Study or Subgroup	Favours Silicone Mean	SD	Total	Hydrophobic Mean	SD	Total	Weight	Std. Mean Difference IV, Random, 95% CI
**1.1.1 Group1 (>=0, <3Y)**								
Findl O 2005	1.6	1.6	56	1.7	1.6	56	9.8%	-0.06 [-0.43, 0.31]
Hayashi K 2001	14.1	9.2	83	11.7	7.6	96	10.5%	0.29 [-0.01, 0.58]
Prosdocimo G 2003	0.089	0.17	40	0.365	0.47	38	8.9%	-0.78 [-1.24, -0.32]
Vock L, Crnej A 2009	1.6	0.7	22	1.7	0.7	22	7.6%	-0.14 [-0.73, 0.45]
**Subtotal (95% CI)**			**201**			**212**	**36.8%**	**-0.15 [-0.61, 0.30]**
Heterogeneity: Tau² = 0.17; Chi² = 14.69, df = 3 (P = 0.002); I² = 80%								
Test for overall effect: Z = 0.66 (P = 0.51)								
**1.1.2 Group2 (>=3, <6Y)**								
Abhilakh Missier KA 2003	2.056	1.415	107	1.178	1.636	107	10.7%	0.57 [0.30, 0.85]
Daynes T 2002	0.42	0.52	43	0.55	0.66	52	9.4%	-0.21 [-0.62, 0.19]
Findl O 2005	1.9	1.6	56	2.2	1.6	56	9.8%	-0.19 [-0.56, 0.19]
Kohnen T 2008	0.0005	0.235	60	0.044	0.289	60	0.0%	-0.16 [-0.52, 0.19]
Vock L, Crnej A 2009	1.9	1.1	22	2	0.9	22	7.6%	-0.10 [-0.69, 0.49]
Wejde G 2004	0.223	0.235	47	0.054	0.289	45	0.0%	0.64 [0.22, 1.06]
Zemaitiene R 2011	0.158	0.194	30	0.171	0.208	31	8.5%	-0.06 [-0.57, 0.44]
**Subtotal (95% CI)**			**258**			**268**	**46.0%**	**0.02 [-0.35, 0.40]**
Heterogeneity: Tau² = 0.13; Chi² = 16.74, df = 4 (P = 0.002); I² = 76%								
Test for overall effect: Z = 0.13 (P = 0.90)								
**1.1.3 Group3 (>=6Y)**								
Vock L, Crnej A 2009	2.3	1.4	22	3.8	2	22	7.4%	-0.85 [-1.47, -0.23]
Vock L 2009	1.4	2.6	44	2.76	2.78	99	9.9%	-0.50 [-0.86, -0.14]
**Subtotal (95% CI)**			**66**			**121**	**17.2%**	**-0.59 [-0.90, -0.28]**
Heterogeneity: Tau² = 0.00; Chi² = 0.95, df = 1 (P = 0.33); I² = 0%								
Test for overall effect: Z = 3.69 (P = 0.0002)								
**Total (95% CI)**			**525**			**601**	**100.0%**	**-0.16 [-0.43, 0.11]**
Heterogeneity: Tau² = 0.16; Chi² = 48.07, df = 10 (P < 0.00001); I² = 79%								
Test for overall effect: Z = 1.15 (P = 0.25)								
Test for subgroup differences: Chi² = 6.54, df = 2 (P = 0.04), I² = 69.4%								

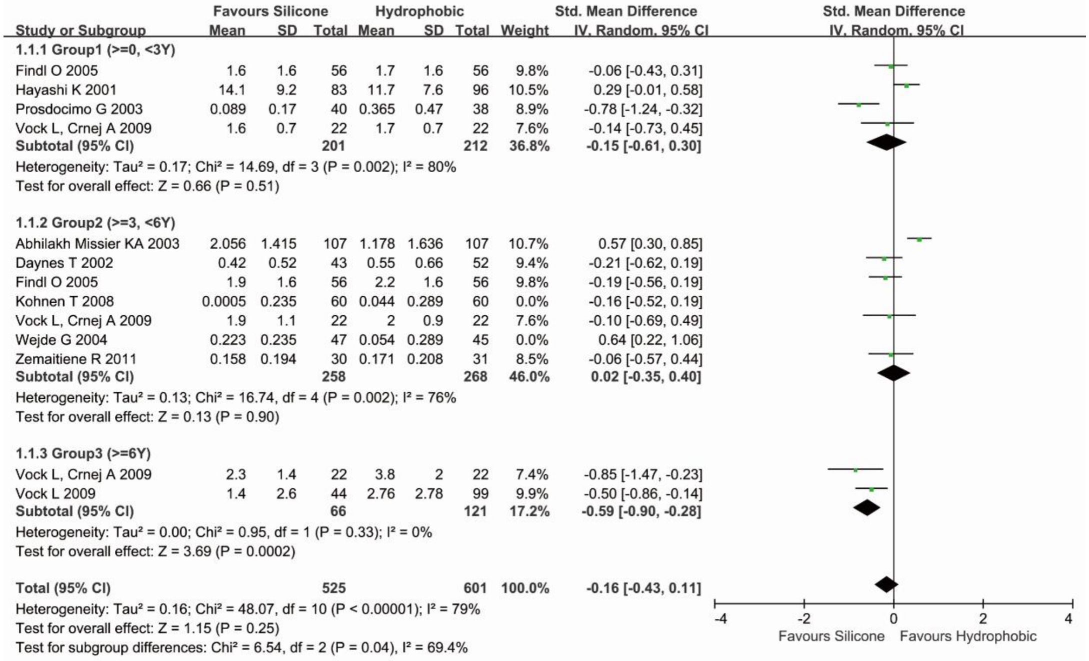

b. The overall effect of ACO value (Chi^2 = chi-square statistic, CI = confidence interval, df = degrees of freedom, I^2 = I-squared, heterogeneity statistic, IV = inverse variance, SMD = standard mean difference, Z = Z-statistic).

Study or Subgroup	Silicone Mean	SD	Total	Hydrophobic Mean	SD	Total	Weight	Std. Mean Difference IV, Random, 95% CI
Daynes T 2002	0.59	0.46	43	0.32	0.3	52	34.0%	0.70 [0.29, 1.12]
Findl O 2005	19.5	8	56	19.7	8	56	0.0%	-0.02 [-0.40, 0.35]
Sacu S 2006	0.2	0.08	53	0.21	0.08	53	34.8%	-0.12 [-0.51, 0.26]
Zemaitiene R 2011	2.333	0.758	30	1.58	0.886	31	31.2%	0.90 [0.37, 1.43]
**Total (95% CI)**			**126**			**136**	**100.0%**	**0.48 [-0.16, 1.12]**
Heterogeneity: Tau² = 0.27; Chi² = 12.75, df = 2 (P = 0.002); I² = 84%								
Test for overall effect: Z = 1.46 (P = 0.14)								

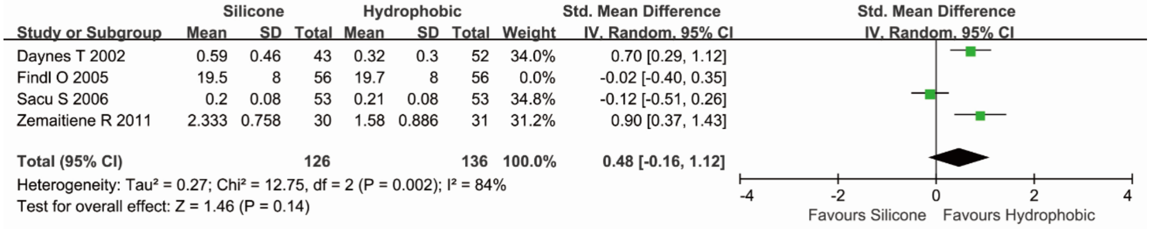

Supplementary File 9. Funnel plots of publication bias

a. Subgroup analysis effects of PCO value (SE = standard error, SMD = standard mean difference).

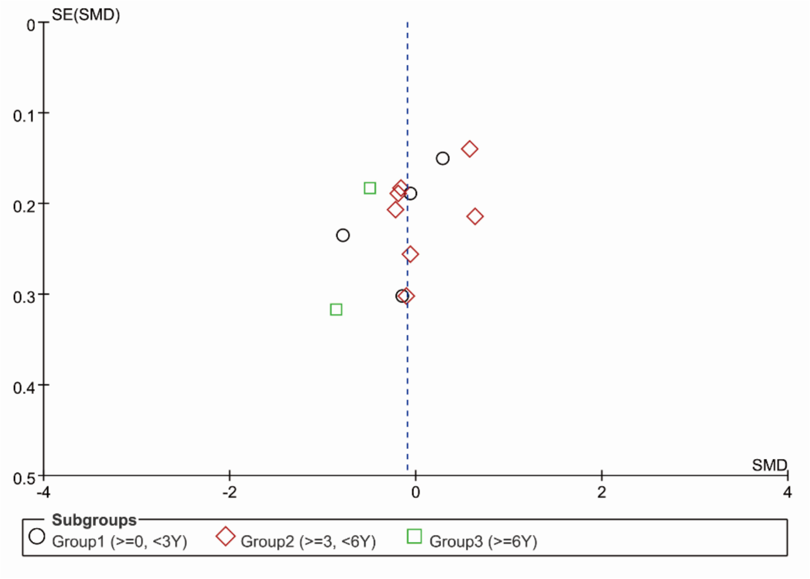

b. Subgroup analysis effects of Nd:YAG capsulotomy rate (SE = standard error, RR = risk ratio).

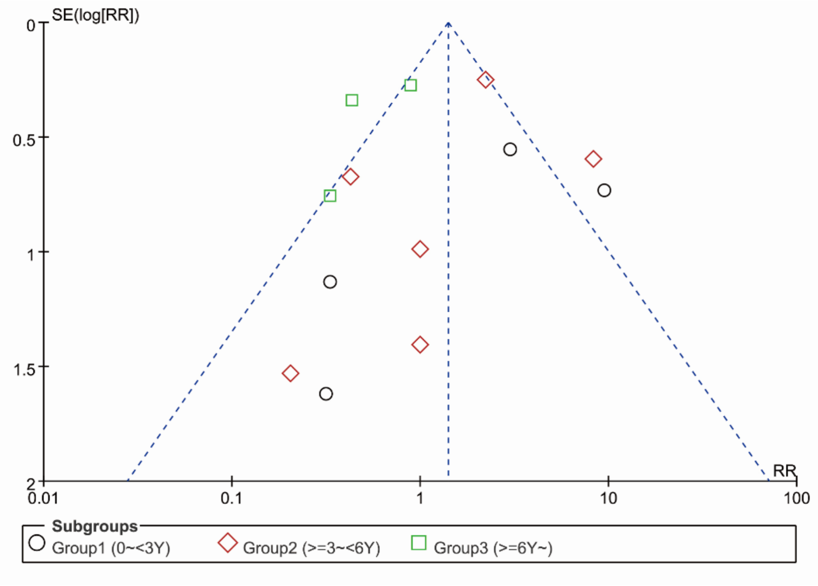

c. The overall effect of ACO (SE = standard error, SMD = standard mean difference).

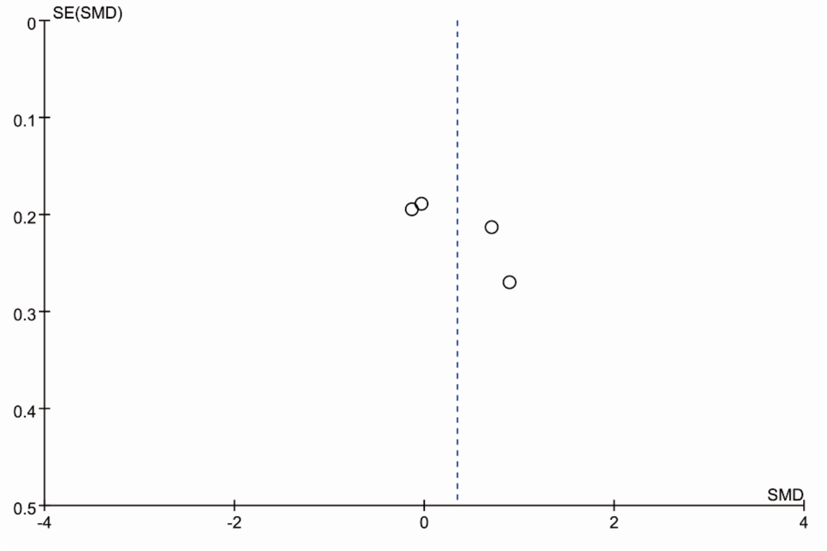

d. The overall effect of visual acuity (SE = standard error, MD = mean difference).

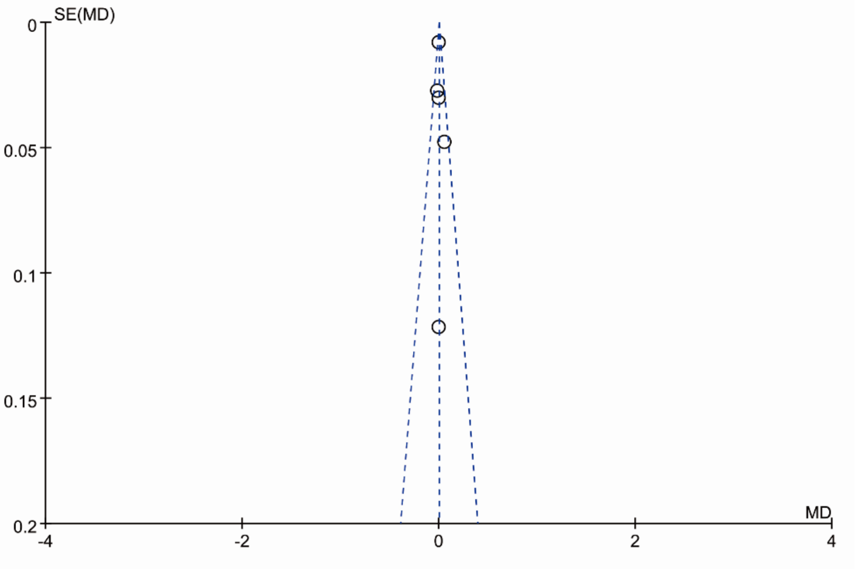

e. The overall effect of tilt (SE = standard error, MD = mean difference).

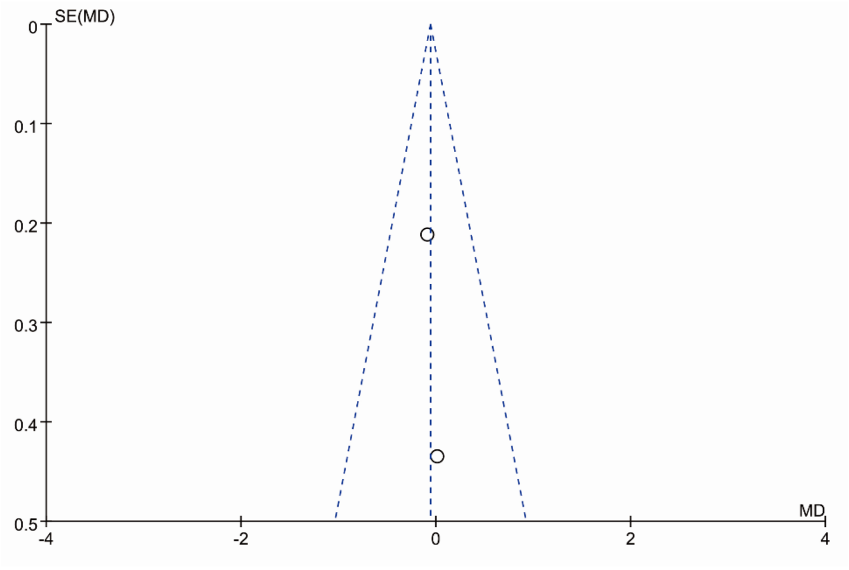

f. The overall effect of decentration (SE = standard error, MD = mean difference).

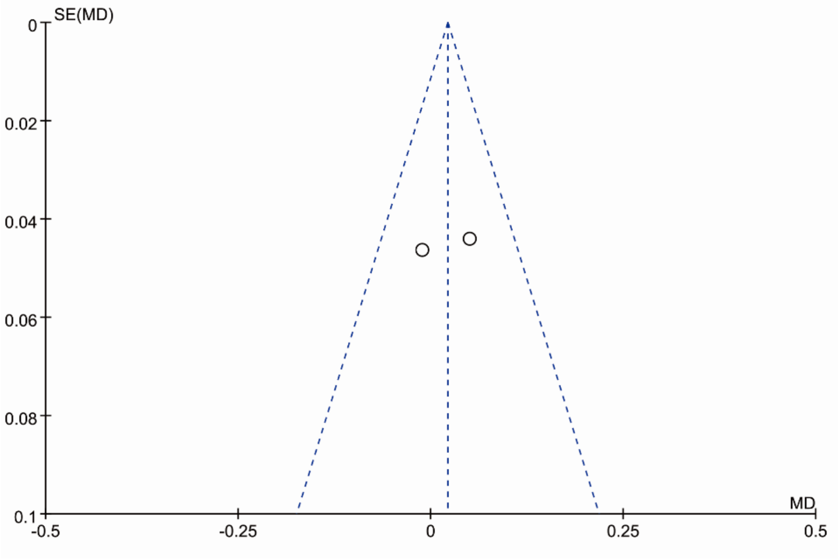

Supplement: Supplementary Materials — Supplementary File 1. PRISMA 2020 checklist. Supplementary File 2. Details of the search strategy. Supplementary File 3. Details of excluded studies and reasons. Supplementary File 4. Characteristics of IOLs included studies. Supplementary File 5. Risk of bias assessment of randomized controlled trials. Supplementary File 6. Risk of bias assessment of nonrandomized controlled trials. Supplementary File 7. Forest plots of the overall effect of PCO value and Nd : YAG capsulotomy rate. Supplementary File 8. Sensitivity analysis except the studies missing SDs. Supplementary File 9. Funnel plots of publication bias. [file 3570399.f1.pdf]
